# Bowel Ischemia in a Patient With SARS CoV-2-Like Illness and Negative Real-Time Reverse Transcription Polymerase Chain Reaction Test Results During the Peak of the Pandemic

**DOI:** 10.7759/cureus.10442

**Published:** 2020-09-14

**Authors:** Ibrahim Almafreji, Sathyaprakash Ranganath

**Affiliations:** 1 Trauma and Orthopaedics, University Hospital Crosshouse, Kilmarnock, GBR; 2 Emergency Department, University Hospital Ayr, Ayr, GBR

**Keywords:** sars-cov-2 (severe acute respiratory syndrome coronavirus -2), covid-19, bowel ischemia, rt-pcr (real time - reverse transcription polymerase chain reaction), coronavirus pandemic, atypical covid-19, personal protective equipment (ppe), thromboembolic disease

## Abstract

An 83-year-old man presented to the emergency department (ED) during the peak of the first wave of the SARS CoV-2 (COVID-19) pandemic with severe abdominal pain, mimicking a severe abdominal pathology. He was found to have features suggestive of COVID-19 infection radiologically, with no leaking aortic aneurysm, bowel ischemia, pancreatitis, or perforation. With worsening symptoms, a repeat computer tomography (CT) scan four days later showed features of bowel ischemia, and he underwent a laparotomy and right hemicolectomy. Four real-time reverse transcription-polymerase chain reaction (rRT-PCR) tests were negative. He was still considered to be infected with COVID-19 and died from complications arising from multi-organ failure. This case highlights an atypical presentation of a possible COVID-19 infection, the urgency to have additional diagnostic tests apart from rRT-PCR, and the necessity to use the appropriate personal protective equipment (PPE) during the pandemic.

## Introduction

The SARS CoV-2 (COVID-19) infection is an ongoing global health crisis. As of July 31, 2020, 359,537 tests were carried out in Scotland. A total of 18,627 results returned positive for COVID-19, with 255 of these cases currently in hospital. Four patients were in-patients in intensive care units (ICUs) with suspected or confirmed COVID-19. In the Ayrshire and Arran counties, there have been a total of 1,275 positive cases so far, with less than 5 cases currently in hospital. Currently, the total number of deaths in Scotland is 2,491, whilst in the Ayrshire region, it is 295 [1].

The disease most commonly presents with respiratory symptoms and/or pyrexia [2]. However, there have been increasing numbers of atypical presentations [3-4]. We report a case, initially presenting with severe abdominal pain, with the early radiological features of possible COVID-19 infection and delayed features of ischemic bowel, which created diagnostic and management dilemmas. As the initial presentation was not typical for COVID-19 infection, the ambulance and medical personnel attending the patient were not wearing appropriate personal protective equipment (PPE).

As of early March 2020, the preferred method of testing for COVID-19 infection in Scotland is the detection of viral ribonucleic acid (RNA) (antigen) in samples, using real-time reverse transcription-polymerase chain reaction (rRT-PCR) [5]. Samples from University Hospital Ayr are sent to the centralized microbiology laboratory at NHS (National Health Service) Ayrshire and Arran’s University Hospital Crosshouse and to the Specialist Virology Centers in Glasgow and Edinburgh. These centres choose polymerase chain reaction (PCR) platforms depending on the current availability of PCR reagents. The accuracy of these tests is dependent on the proper technique used to obtain samples, especially from the throat and nasal swabs. Also, viral RNA on these swabs may degrade over time while being transported to designated testing centres. At present, other antigen and antibody detection tests via serology have not been utilized in Scotland and the United Kingdom (UK).

## Case presentation

An 83-year-old-male presented to the emergency department (ED) at University Hospital Ayr in mid-April, by ambulance, with severe abdominal pain radiating to his back and diaphoresis. This prompted the ambulance crew to administer intravenous (IV) morphine. They specifically reported that he did not have any symptoms of COVID-19 infection (fever, cough, breathlessness, or requiring oxygen) and hence was shifted to the non-COVID area in resuscitation. In addition to severe abdominal pain, he complained of 2 days history of nausea and a few episodes of loose stools.

His past medical history included ischemic heart disease, type 2 diabetes mellitus, hypertension, chronic kidney disease stage 3, atrial fibrillation, and prostate cancer for which he received radiotherapy and neoadjuvant hormonal therapy in 2012. His regular medications included furosemide 40 milligrams (mg), clopidogrel 75 mg, metformin 500 mg, bisoprolol 5 mg, doxazosin 4 mg once daily, and gliclazide 40mg twice daily.

On examination, the patient was still experiencing unbearable pain on a scale of 10/10 and was sweating profusely. His temperature was 36° centigrade, heart rate 85 per minute, blood pressure 195/80 mmHg, respiratory rate 16/minute, and oxygen saturation 95% on air. He was alert and orientated. On auscultation of his chest, there was good air entry bilaterally with normal breath sounds and no crepitation, wheeze, or rhonchi. His abdomen was soft but distended with generalized tenderness, maximal in the right iliac fossa. There was no guarding or rebound tenderness. There was no palpable or pulsatile mass. His bowel sounds were normal. The rest of his examination was unremarkable. He required another two 5 mg boluses of IV morphine for pain control.

Investigations

On admission to the ED, the patient’s blood tests demonstrated metabolic acidosis, hyperglycemia, and raised inflammatory markers, high lactate, and lymphopenia (Table 1). Amylase was normal, hence ruling out pancreatitis. Electrocardiogram (ECG) showed rate-controlled atrial fibrillation with no new ST-segment changes.

**Table 1:** Blood Test Results This table illustrates the trend in the patient's blood test results over the course of his admission.

Test (Normal values)	Day 1	Day 4	Day 6	Day 7	Day 9
Hydrogen ions (35-45 nmol/l)	50.8	52.3	50	63.6	60.6
Bicarbonate (22-29 mmol/l)	18.2	22.1	21	15.4	16.8
Glucose (3.2-6.1 mmol/l)	22.17	6.7	6.2	9.1	5.1
Lactate (0.7-2 mmol/l)	5.28	1.22	1.3	1	1.01
White cell count (3.7-9.5 x109g/l)	15.9	13.4	9.7	13.5	11.3
Neutrophils (1.5-6.5 x109g/l)	15.2	12.1	8.8	12.9	10.8
Lymphocytes (1.1-5 x109g/l)	0.3	0.5	0.4	0.3	0.2
C-reactive protein (2-10 mg/l)	139	406	356	421	250
Urea (2.5-7.5 mmol/l)	10.9	18	20.2	22.3	28.9
Creatinine (50-125 mmol/l)	126	177	228	277	283
Estimated glomerular filtration rate (>60 ml/min)	49	33	24	19	19
Amylase (20-100 U/l)	37	-	-	-	-
D-dimers (0-500 ng/ml)	2959	-	-	-	-
Troponin T (0.01-0.03 µg/L)	0.202 (12-hour repeat - 0.358)	-	0.48	-	-
Ferritin (30-400 ug/l	150	-	-	-	-

**Figure 1:**
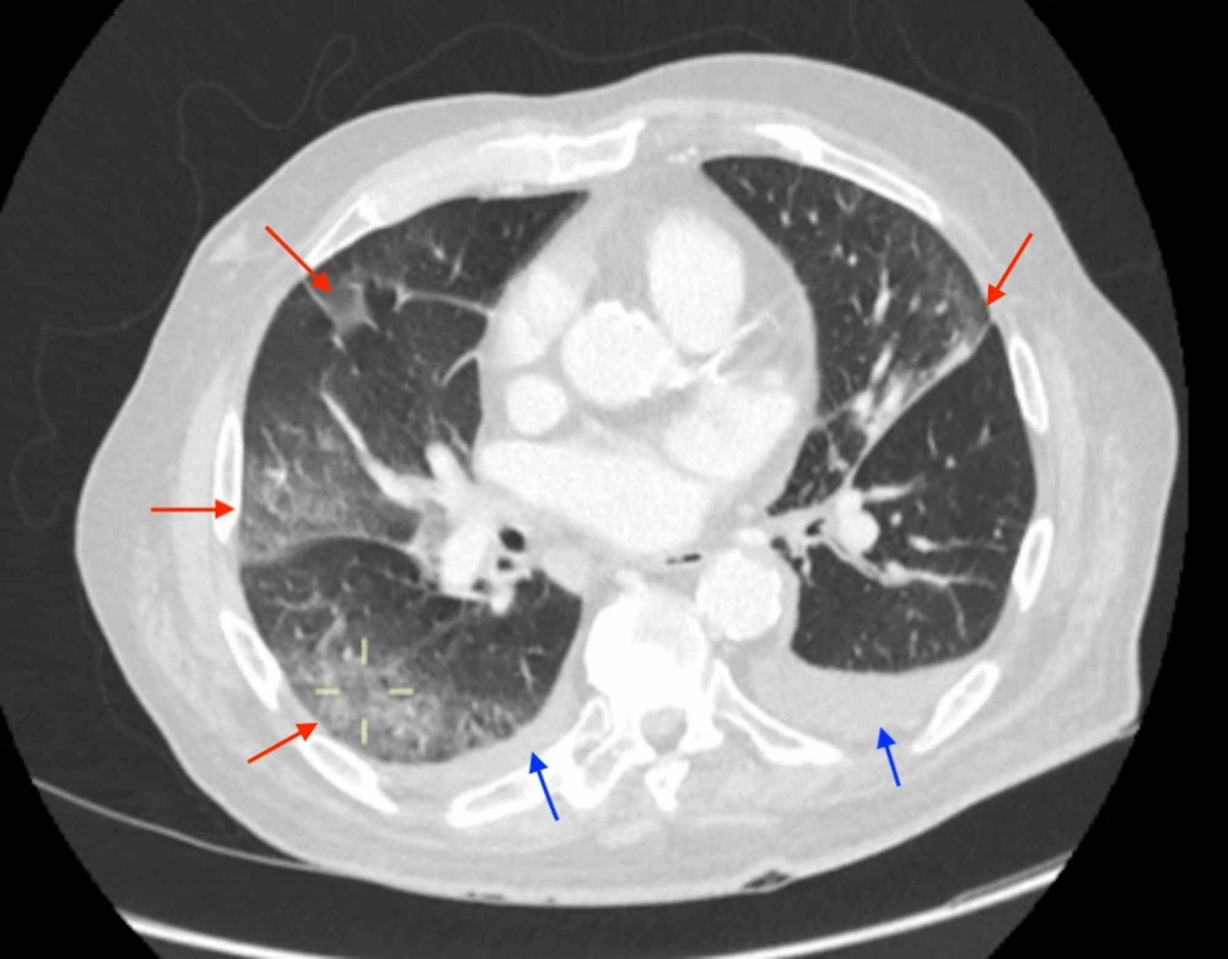
CT Chest - Axial View Peripheral ground-glass opacification within the right upper and lower lobes and the left upper lobe (red arrows). Minimal changes were noted within the lingula. There were small bilateral pleural effusions (blue arrows). These appearances were considered equivocal for COVID-19 infection.

**Figure 2:**
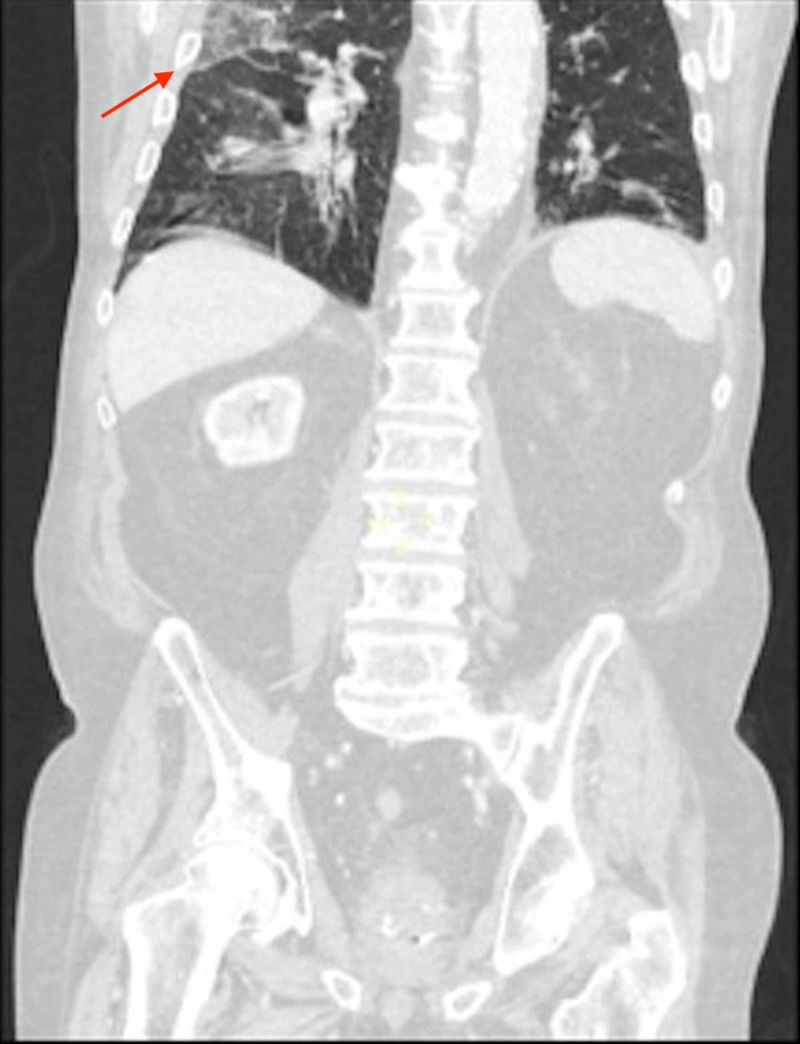
CT - Coronal View Peripheral ground-glass opacification within the right upper and lower lobes (red arrow) on coronal view. Combined with the findings on the axial view, these appearances were considered equivocal for COVID-19 infection.

**Figure 3:**
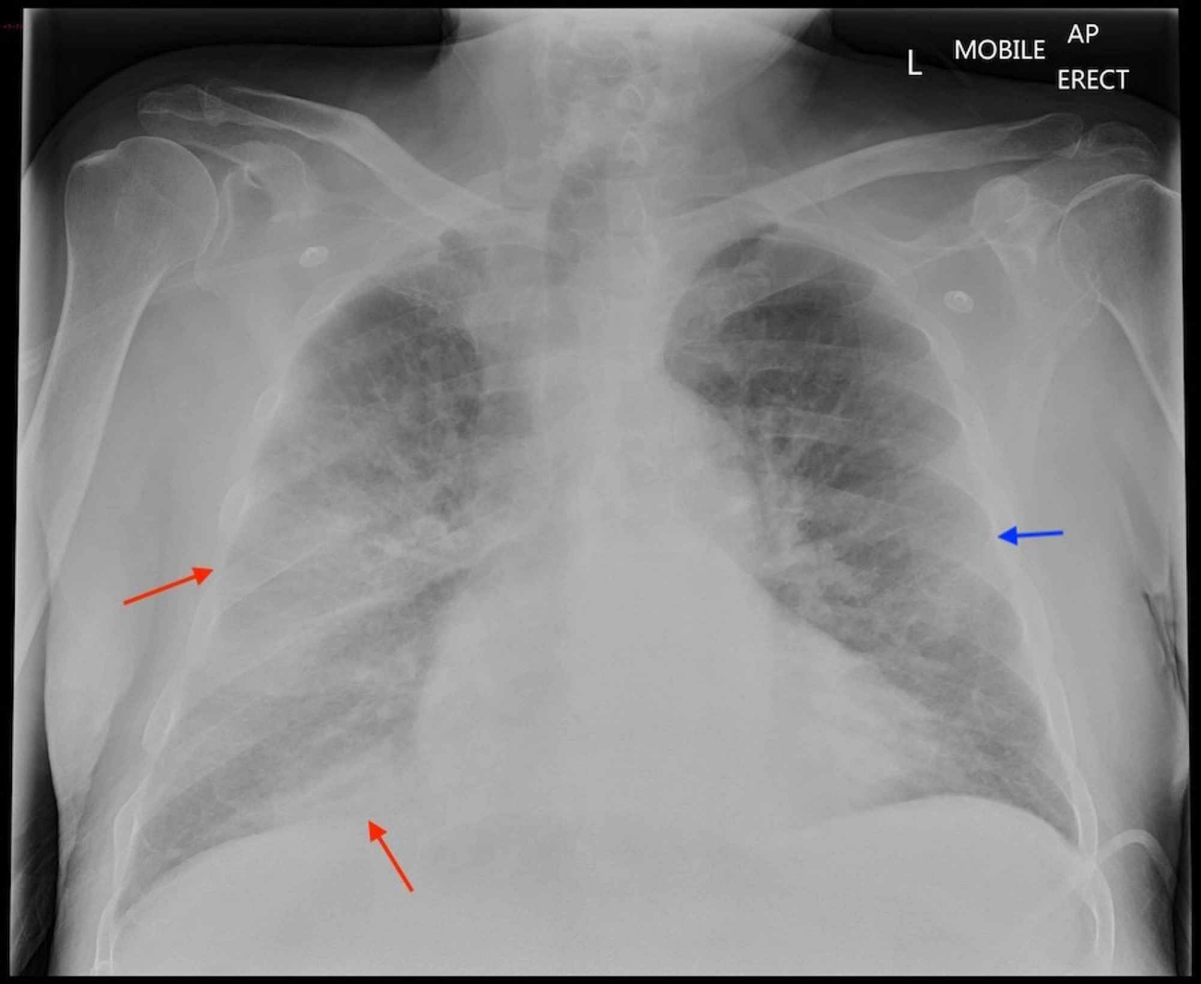
CXR - AP view Ground glass opacification and patchy airspace shadows are seen peripherally in the right middle and lower lung zones (red arrows) and increased attenuation seen peripherally in the left middle lung zone (blue arrow). These features were suspicious of COVID-19 pneumonia. CXR: Chest X-ray; AP: Anteroposterior

A computed tomography (CT) scan of his chest, abdomen, and pelvis in the arterial and venous phases was carried out to rule out life-threatening abdominal pathology. This demonstrated diffuse aortoiliac calcification, but the visceral arteries appeared grossly patent. There were no features of aortic aneurysm, bowel ischemia, obstruction, perforation, or pancreatitis. Incidentally, there were peripheral ground-glass opacities within the right middle and lower lobes of both lungs and minimal changes within the lingula. There were also small bilateral pleural effusions (Figures 1-2). These appearances were considered by the radiologist as equivocal for COVID-19 infection. A chest X-ray (CXR) showed bilateral pulmonary ground-glass opacification suggestive of COVID-19 infection (Figure 3).

He was reviewed by the surgical team while in the ED. As there was no evidence of mesenteric ischemia or colitis on his initial CT scan, they referred the patient to the acute medical team to manage him for potential COVID-19 infection, hyperglycemia, and metabolic acidosis. He was managed in the COVID ward. Blood tests for serum troponin T, ferritin, and D-dimers were sent as part of the COVID-19 investigation protocol of the hospital (Table 1). They demonstrated raised d-dimers and troponin T values, the latter indicating cardiac involvement.

On Days 1 and 2 of admission, two swabs were taken from the nose and throat, which were transported to the NHS Ayrshire and Arran centralized laboratory for rRT-PCR testing. This laboratory is using the Abbott M2000 (Abbott Molecular, Chicago, Illinois) real-time reverse transcriptase SARS CoV-2 PCR platform, with a turnover time of seven hours. Both test results were negative. We got results in approximately 12 hours. This included the transit time for the samples to reach the centralized laboratory in another hospital. Despite the results of the negative swabs, in view of the limitations of this test, precautions for COVID-19 infection remained, as he was highly suspicious of being infected with the virus. The manufacturer of this test, Abbott Molecular, has acknowledged the limitations of this test specifying that the detection of SARS-CoV-2 RNA may be affected by the (1) Sample collection methods, patient factors (e.g.: the presence of symptoms), and/or stage of infection; (2) Degradation of the viral RNA during shipping/storage; (3) Mutations within the target regions of SARS-CoV-2 assays, which would affect the primer and probe binding [6].

On the fourth day of admission, he complained of worsening generalized abdominal pain despite regular analgesia. Clinically, his abdomen was more distended, tense, and generally tender, signifying peritonitis. His blood tests showed worsening inflammatory markers and renal function (Table 1). A repeat CT scan demonstrated an irregular appearance of ascending colon suggestive of colitis, possibly due to ischemia. During laparotomy, ischemic ascending colon and terminal ileum were confirmed.

On the sixth day of admission, 12-lead ECG showed a new right bundle branch block and blood tests showed a troponin T of 0.476 µg/L, further signifying cardiac involvement. Another nasopharyngeal swab and a sample of tracheal aspirates were sent to the specialist virology centre for SARS CoV-2 rRT-PCR tests. Both results came back negative.

By the seventh day, his inflammatory markers and renal function worsened. This trend continued until his last day (Table 1). Echocardiogram (2D-ECHO) on the eighth day of admission showed bi-atrial dilatation and mild-moderate left ventricular (LV) systolic impairment (2D-ECHO in 2017 showed normal LV function). The ejection fraction was calculated at 45%.

The histopathology of the resected ascending colon and terminal ileum was reported three days after the patient’s death. It demonstrated extensive mucosal ulceration, congestion with areas of extensive transmural inflammation, and transmural infarction. Occasional fibrin thrombi were noted in some of the blood vessels in the submucosa at the areas of ulceration. Overall, these features were in keeping with ischemia and peritonitis.

Differential diagnosis

In the emergency department, due to acute abdominal pain, raised lactate, the background of ischemic heart disease, and atrial fibrillation, the differential diagnoses were a leaking abdominal aortic aneurysm, ischemic bowel/mesenteric embolism, severe pancreatitis, or bowel perforation. These were ruled out on the initial CT scan. In view of the atypical lung findings (Figures 1-2), COVID-19 infection was suspected.

On admission to the ward, teams from the acute medicine, surgery, and anaesthesia also strongly suspected the patient to be infected with COVID-19 due to the atypical pulmonary infiltrates on CXR and CT scan. The initial two nose-throat swabs were negative for COVID-19 virus RNA.

A repeat CT scan of the abdomen a few days later suggested the presence of bowel ischemia. This was confirmed by the surgeons during surgery and later by the pathologist.

A repeat CXR while in the ICU still showed persistent atypical pulmonary changes/infiltrates. After reviewing the patient and the radiological images, the microbiologist suggested that the patient may be having micro-thrombotic infiltrates in the lungs similar to COVID-19 infection. Another nose-throat swab and a tracheal aspirate to test for COVID-19 infection did not detect the viral RNA.

The patient developed worsening cardiac and renal functions, as indicated by 2D-ECHO and blood results. Failure to extubate the patient, with no respiratory drive, suggested decreased brain function. This indicated that he had developed multi-organ failure.

Treatment

In the emergency department, the patient received IV crystalloids for metabolic acidosis, hyperglycemia, and dehydration. He was commenced on antibiotics to treat lung infection and received analgesics (Table 2).

**Table 2:** Medications Received by the Patient During His Admission ICU: Intensive Care Unit; IV: Intravenous; 1g: 1 gram; MG: Milligram; QDS; quater die sumendum (to be taken four times daily); TDS: ter die sumendum (to be taken three times daily); BD: bis die sumendum (two times daily); OD: omne in die (once daily); LMWH: Low Molecular Weight Heparin; SC: Subcutaneous

Days in hospital	1	1 to 4	5	6	7 and 8	9
Department	Emergency Department	COVID Ward	ICU	ICU	ICU	ICU
IV Fluids	Normal saline 0.9%	Normal saline 0.9%	Normal saline 0.9%	Normal saline 0.9%	Stopped	-
Analgesia	IV morphine-titrated, IV paracetamol 1g qds	IV paracetamol 1g qds, oral opiates-titrated	IV paracetamol 1g qds	IV paracetamol 1g qds	IV paracetamol 1g qds	IV paracetamol 1g qds
1^st^ antibiotic regimen	IV amoxicillin 1g tds and oral clarithromycin 500mg bd	IV amoxicillin 1g tds and oral clarithromycin 500mg bd	-	-	-	-
2^nd^ antibiotic regimen	-	-	IV amoxicillin 1g tds, IV metronidazole 500mg tds and IV gentamycin 240mg od	IV amoxicillin 1g tds, IV metronidazole 500mg tds and IV gentamycin 240mg od	IV amoxicillin 1g tds, IV metronidazole 500mg tds and IV gentamycin 240mg od	-
Oxygen delivered	-	Nasal	Ventilator	Ventilator	Ventilator	Mask
Prophylactic LMWH	-	SC dalteparin 5000 units od	Stopped for surgery	SC dalteparin reduced dose 2500 units od	SC dalteparin reduced dose 2500 units od	SC dalteparin reduced dose 2500 units od
Inotropes	-	-	Noradrenaline infusion	Noradrenaline infusion	Noradrenaline infusion	Noradrenaline infusion tapered and stopped
Diuretic	-	-	-	-	IV furosemide 1mg/ hour	IV furosemide 1mg / hour
Steroids	-	-	-	-	IV hydrocortisone 50 mg qds	IV hydrocortisone 50mg qds

In the COVID ward, his oxygen saturation dropped to 88% on room air and the required supplemental oxygen for the first five days until he was intubated. Metformin was stopped due to high lactate. He received oral opiate analgesia to manage pain. Prophylactic low molecular weight heparin (LMWH) was given to prevent venous thrombosis. On the fifth day of admission, after a repeat CT scan, the antibiotic regimen was changed to treat the intra-abdominal sepsis (Table 2).

He was intubated and mechanically ventilated prior to surgery. He underwent a right hemicolectomy and resection of the terminal ileum with ileocolic anastomosis and was transferred to the ICU.

In the ICU, his mechanical ventilation was continued and he required inotrope support. The LMWH dose was reduced due to worsening renal functions. He continued to receive IV antibiotics and crystalloids (Table 2).

On Day 7, his crystalloids were stopped due to fluid overload from renal and heart failures. A diuretic infusion was started. To regulate the inflammatory response steroid injection was commenced. He received them until palliation on Day 9 (Table 2).

Despite the treatment, the patient had persistent metabolic acidosis (Table 1). In the ICU, he developed multi-organ failure. He did not make any significant recovery and required continuous ventilatory and inotropic support. He failed two attempts at extubation with no respiratory drive. The decision was made by the teams and family to stop all active treatment and make him comfortable. The patient died nine days after admission. A post-mortem examination was not done.

## Discussion

COVID-19 infection continues to present in a variety of ways. The majority of cases were asymptomatic, according to the data from China [7]. Although pyrexia, cough, and fatigue were the most common initial symptoms [2], atypical presentations have been demonstrated [3-4]. Gastrointestinal involvement, such as abdominal pain and diarrhoea, similar to our case, has been reported in the literature [3].

The patient described in this case report did not exhibit any respiratory symptoms initially. We did not suspect COVID-19 infection until ground-glass opacities (GGO) in the lungs, suggestive of the disease, were incidentally found on the CT scan [8]. This delay resulted in the exposure of our health care workers without appropriate PPE.

A variety of CT findings has been reported related to COVID-19 [8]. The main features of COVID -19 pneumonia are the presence of opacities with ground glass appearance (GGO), typically in peripheral and subpleural areas, involving multiple lobes in both lungs, especially the lower lobes [8]. A study of 1014 patients in China reported that the sensitivity of CT scans is 97%, as compared to the gold standard reverse-transcription polymerase chain reaction (RT-PCR) test [9].

RT-PCR is currently the gold standard test for diagnosing COVID-19. In this test, viral RNA is converted to DNA by reverse transcription, and then, the DNA is amplified using the polymerase chain reaction, which is then analyzed. However, there have been cases of false-negative RT-PCR results in the early and later stages of the disease. This could be due to inadequate viral material in the sample or technical issues during nucleic acid extraction [10]. According to our centralized test centre, the sensitivity of the test is 60%-80% [11]. Therefore, a false-negative test was a strong consideration in our case. According to Watson J et al., the approximate sensitivity and specificity of available RT-PCR tests are 70% and 95%, respectively [12]. We should use clinical judgment and decide whether to continue COVID-19 precautions for the patient and health care worker’s safety. This has been suggested in articles by Abbott Molecular and Graziadio S et al. [6,13]. In this case, the patient was continuously isolated in a COVID-19 area while health care workers wore the appropriate PPE despite negative initial rRT-PCR results, as he had clinical signs of COVID-19 type infection involving multiple organs.

Esbin MN et al. provides a review of the current nucleic acid testing approaches. Alongside the gold standard RT-PCR test, he presents other promising methods, such as isothermal amplification, Clustered regularly interspaced short palindromic repeats (CRISPR)-based detection, which can help achieve faster and more accurate results [14]. Bastos ML et al. review current serological testing and reports that the existing evidence was characterized by a high risk of bias, heterogeneity, and limited generalizability to point-of-care testing and to outpatient populations. Pooled sensitivities were also seen to be lower in commercial kits and in the first and second weeks after symptoms onset. It concludes that evidence does not support the continued use of existing point-of-care serological tests for COVID-19 [15].

There is no specific treatment or cure for COVID-19. The current treatment protocol for COVID-19 infection in the Ayrshire and Arran NHS Hospitals is centred around general supportive care, treatment for underlying conditions, and antibiotics for secondary bacterial infections. In-patients who test positive for COVID-19 are recruited for national trials. The Randomised Evaluation of COVID-19 Therapy (RECOVERY) trial allocates patients to one of six treatments: lopinavir-ritonavir, low-dose dexamethasone, hydroxychloroquine, azithromycin, tocilizumab, and convalescent plasma [16]. This patient was not recruited for the aforementioned trials, as they had not been locally implemented at the time of the patient’s hospital admission.

Our patient received low-dose oxygen, prophylactic dose LMWH, IV fluids, and antibiotics to cover bacterial pneumonia and abdominal infections. The mechanical ventilation was for the induction of general anaesthesia during surgery and not for respiratory failure. He was given IV steroids later on, during his admission.

Thromboembolic complications of COVID-19 have been reported. These are mostly pulmonary and it has not been proven whether this is due to COVID-19 or the effect of a cytokine storm [17]. Lia a Beccara L et al. reported a case of arterial mesenteric thrombosis as a complication of COVID-19 [18]. This suggests that systemic micro-embolization must be considered in addition to pulmonary thrombotic complications. Some clinicians use intermediate or therapeutic dose LMWH, hypothesizing that it could prevent microvascular thrombi [17,19]. There is limited evidence to suggest a benefit from such a practice and warrants further investigation [17]. It is recommended that all these patients are at least on prophylactic dose LMWH, according to local policies. There is no definitive guidance on the risk stratification for thromboembolic disease and dosages of LWMH [20].

We believe that this patient had developed a viral illness two days prior to his attendance at ED. Initially, this had spread to his lungs and later involved the bowels and other organs with systemic micro-embolization causing multi-organ failure. Even though we could not confirm the diagnosis of COVID-19 infection with the available rRT-PCR test, the entire clinical presentation suggests otherwise.

## Conclusions

COVID-19 infection presents in numerous ways, and patients may not present with respiratory symptoms. Thus, we must maintain a high level of suspicion. Health care workers also need to be vigilant of atypical presentations of COVID-19 infection and consider wearing appropriate PPE for all patients during the pandemic. If our patient was infected with COVID-19, the inflammatory cascade, which is known to cause thromboembolic phenomena, might have caused micro-emboli to the mesenteric blood supply causing bowel ischemia. Serological tests have been reported to have disadvantages and are unreliable for diagnosis. Sampling errors, delays in testing the samples, and other limitations should be considered while treating a suspected COVID-19 patient whose rRT-PCR test is reported negative. Further research and development are required in nucleic acid testing in order to produce faster and more accurate diagnostic tests.
